# Diverse Forms of Autophagy and Their Roles in Liver Disease and Aging: A Comprehensive Review

**DOI:** 10.3390/proteomes14020028

**Published:** 2026-05-27

**Authors:** Seoyoon Heo, Min Young Lee, Che Yeon Jeong, Dong Ha Kim, Ji Hye Jun

**Affiliations:** Division of Life Science, College of Natural Sciences, Gyeongsang National University, Jinju 52828, Republic of Korea; hsy040813@naver.com (S.H.); 2021010598@gnu.ac.kr (M.Y.L.); cheayeon0929@naver.com (C.Y.J.); tjdrms011103@naver.com (D.H.K.)

**Keywords:** selective autophagy, hepatocyte senescence, MASLD, quantitative proteomics, proteoforms, organelle proteome remodeling

## Abstract

The liver is a central metabolic organ that integrates nutrient sensing, lipid handling, and detoxification to maintain systemic homeostasis. In metabolic dysfunction–associated steatotic liver disease (MASLD), chronic metabolic overload accelerates hepatocyte senescence, impairing regenerative capacity and promoting progression toward fibrosis and hepatocellular carcinoma. While transcriptomic studies have provided important insights into stress-responsive pathways, they incompletely capture the proteome remodeling and proteoform-level alterations that govern hepatocyte function during aging and disease. Recent mass spectrometry–based proteomics studies have revealed that disruption of autophagy-dependent proteome homeostasis is a defining feature of senescent hepatocytes. Quantitative analyses demonstrate coordinated alterations in selective autophagy pathways—including lipophagy, mitophagy, ferritinophagy, ER-phagy, and pexophagy—accompanied by organelle-specific protein abundance signatures and remodeling of autophagy-related proteoforms. These findings position proteomics as an essential tool for resolving the spatial and functional reorganization of hepatocyte proteomes that cannot be inferred from transcript abundance alone. In this review, we synthesize proteomics-driven evidence defining selective autophagy dysfunction in aging and MASLD livers, critically evaluate methodological limitations, and propose a conceptual framework in which impaired selective autophagy acts as a proteome-level driver of hepatocyte senescence. We further outline future directions for proteoform-resolved and spatial proteomics approaches aimed at identifying actionable targets for therapeutic intervention in liver disease.

## 1. Introduction

The liver is the principal metabolic organ in the human body, responsible for a wide array of physiological functions, including energy regulation, detoxification, and protein synthesis. These functions are primarily executed by hepatocytes, whose physiological homeostasis plays a pivotal role in systemic metabolic control. However, as aging progresses, the regenerative capacity and metabolic efficiency of hepatocytes gradually decline, thereby promoting sustained metabolic stress and intercellular dysfunction within the hepatic microenvironment. Therefore, the accumulation of aged hepatocytes has been reported to influence the onset and progression of non-alcoholic fatty liver disease and viral hepatitis, thereby promoting liver injury, inflammation, fibrosis, and hepatocellular carcinoma [1]. Increasing evidence suggests that senescent hepatocytes are not merely a consequence of liver injury but actively drive disease progression through proteome remodeling and altered organelle quality control.

In particular, Metabolic Dysfunction-Associated Steatotic Liver Disease (MASLD) is commonly accompanied by obesity and is closely associated with metabolic syndrome. Hepatic lipid accumulation typically triggers lipotoxicity and mediates MASLD or its progression to Metabolic Dysfunction-Associated Steatohepatitis (MASH) through mechanisms such as endoplasmic reticulum (ER) stress, oxidative stress, organelle dysfunction, and ferroptosis [2]. Importantly, progression from simple steatosis (MASLD) to inflammatory and fibrotic MASH is closely associated with hepatocyte senescence, chronic inflammatory signaling, and progressive collapse of organelle quality control systems. Senescent hepatocytes accumulate in MASH livers and contribute to disease progression through persistent DNA damage response (DDR) signaling, mitochondrial dysfunction, oxidative stress, and senescence-associated secretory phenotype (SASP) production. Increasing evidence further suggests that impairment of selective autophagy pathways–including lipophagy, mitophagy, ferritinophagy, ER-phagy, and pexophagy-plays a central role in driving this transition by promoting proteostasis imbalance and chronic hepatocellular stress. Despite increased fatty acid oxidation, hepatic lipid accumulation is not resolved, and dysfunctional mitochondria generate excessive reactive oxygen species (ROS), thereby contributing to oxidative stress and the development of liver disease [3]. Elevated circulating triglyceride levels promote the influx of free fatty acids (FFA) into non-adipose tissues, leading to ectopic accumulation and dysfunction of non-adipose cells. This intensified lipotoxicity causes significant hepatocyte damage through ER stress, inflammasome activation, and apoptosis [4]. Thus, MASLD serves as a potent accelerator of cellular senescence, establishing a feed-forward cycle between metabolic overload, organelle dysfunction, and proteostasis collapse.

Senescence is a progressive degenerative process occurring from both physiological and pathological perspectives. Physiological senescence refers to the degenerative process following maturation, driven by hallmarks such as telomere attrition, DNA damage, mitochondrial dysfunction, decreased NAD+ (nicotinamide adenine dinucleotide) levels, autophagy impairment, stem cell exhaustion, inflammation, loss of proteostasis, altered intercellular communication, and dysbiosis, all of which culminate in systemic functional decline. Pathological senescence encompasses alterations triggered by various external factors, including cardiovascular disease, cerebrovascular disorders, degenerative joint disease, diabetes, Parkinson’s disease, Alzheimer’s disease, cancer, and the functional deterioration of multiple organs. Importantly, many of these hallmarks converge on disruptions in protein homeostasis and organelle quality control. These regulatory layers are governed predominantly at the level of protein abundance, post-translational modification, multiprotein complex assembly, and proteoform diversity rather than transcript abundance alone. This discrepancy underscores a critical limitation of transcript-centric approaches in defining hepatocyte dysfunction during aging and MASLD progression. These age-related physiological and pathological shifts exert critical impacts on nutrient maintenance, cell signaling, proteostasis, epigenetics, and DNA repair mechanisms [5]. Therefore, investigating age-related regulatory mechanisms is essential for the early detection and treatment of liver disease.

Autophagy is an evolutionarily conserved cellular degradative process that delivers damaged or unnecessarily accumulated organelles to the lysosome for turnover. It plays a vital role in maintaining cellular homeostasis by breaking down proteins, glycogen, lipids, and dysfunctional organelles—such as damaged mitochondria and excessive ER—thereby providing essential nutrients and biomolecular precursors for cell survival. Based on the mode of substrate delivery, autophagy is categorized into three primary types: macroautophagy, microautophagy, and chaperone-mediated autophagy (CMA) [6]. Because autophagy is executed through coordinated multiprotein complexes, dynamic protein–protein interactions, proteoform switching, and organelle-restricted protein networks, mass spectrometry–based proteomics has emerged as an indispensable platform for interrogating its regulation in hepatocytes. Quantitative proteomics enables the detection of coordinated abundance shifts in autophagy-related proteins, pathway-specific organelle signatures, and proteoform-level heterogeneity that cannot be inferred from mRNA profiling alone. These advances position proteomics as a central tool for resolving how selective autophagy pathways—including lipophagy, mitophagy, ferritinophagy, ER-phagy, and pexophagy—are rewired during MASLD progression and hepatocyte senescence.

In this Review, we integrate proteomics-driven evidence to define how selective autophagy dysfunction reshapes the hepatocyte proteome in aging and MASLD. We critically evaluate current methodological limitations, highlight inconsistencies across experimental models, and propose a conceptual framework in which impaired selective autophagy functions as a proteome-level driver of hepatocyte senescence. By reframing autophagy through a proteomics-centered lens, we aim to delineate future research directions that leverage proteoform-resolved and spatial proteomics approaches for therapeutic targeting in liver disease.

## 2. Concepts and Molecular Regulatory Mechanisms of Cellular Senescence

### 2.1. Definition and Characteristics of Cellular Senescence

**Figure 1:**
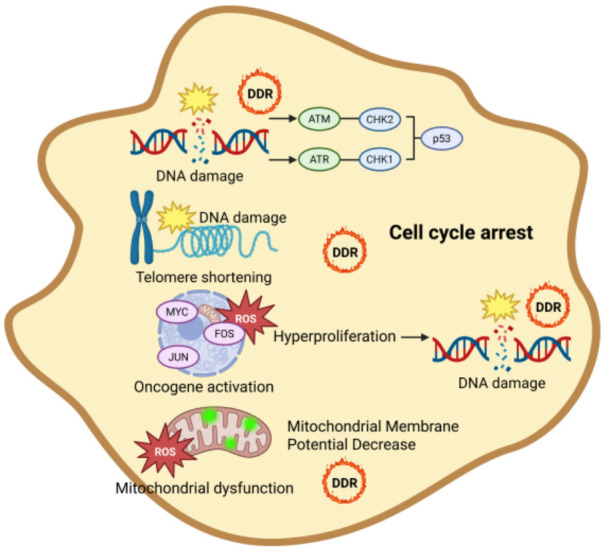
**Diverse Factors Inducing Cellular Senescence, subsequent telomere impairment activate a signaling cascade known as the DNA Damage Response (DDR).** This signaling is transmitted from ATM and ATR to CHK2 and CHK1, ultimately leading to activation of the tumor suppressor protein p53, induction of cell cycle arrest, and, upon prolonged DDR activation, the onset of senescence. Furthermore, the activation of oncogenes promotes hyperproliferation and alterations in DNA replication patterns through the generation of reactive oxygen species (ROS), leading to DNA replication stress and the accumulation of genomic damage. In addition, mitochondrial dysfunction, characterized by low mitochondrial membrane potential (MMP), induces ROS production, which sustains persistent DDR signaling and facilitates the progression of cellular senescence. DDR; DNA damage response, ATM; Ataxia Telangiectasia Mutated, ATR; Ataxia Telangiectasia and Rad3-related, CHK1; Checkpoint Kinase 1, CHK2; Checkpoint Kinase 2, ROS; Reactive Oxygen Species.

Cellular senescence is induced by a variety of factors, including DNA damage, telomere dysfunction, oncogene activation, and organelle-specific stress, and is associated with processes such as tumor suppression, tissue damage repair, and embryonic development. Generally, cellular senescence refers to a terminal state of growth arrest in which cells are unable to proliferate despite optimal mitogenic conditions. Notably, the process of senescence involves overlapping stages ranging from the immediate post-cell cycle exit phase to late-stage senescence, during which multiple molecular pathways act in a coordinated and interconnected manner [7]. Importantly, senescence is not a binary cell state but rather a continuum characterized by progressive remodeling of the cellular proteome, including altered protein abundance, stability, subcellular localization, and post-translational modification patterns (Figure 1).

The phenomenon of senescence was first identified in the 1960s in human diploid cell strains that had exhausted their replicative potential [8]. Senescence is characterized by cell cycle arrest at the G_1_ or G_2_ phases, which prevents the proliferation of damaged cells. In contrast, cellular quiescence, a reversible growth-arrested state induced by nutrient or growth factor deprivation, occurs at the G_0_ phase [9]. Cellular Senescence is characterized by morphological changes regulated by molecular alterations, such as increased p53 expression and inhibition of RB phosphorylation. In general, senescent cells are larger, flatter, and exhibit a more spread morphology than healthy cells [10]. Proteomics-based analyses have revealed that these phenotypic changes are paralleled by coordinated shifts in cytoskeletal, nuclear, and lysosomal protein networks, highlighting the importance of protein-level remodeling in establishing and maintaining the senescent phenotype [11,12].

Senescent cells exhibit abnormal functions and undergo stable cell cycle arrest at the *G*_1_/*G*_2_ phases, rendering them incapable of proliferation. Senescence diminishes the total pool of cells available to perform normal physiological functions within tissues. Under conditions of stress, the rate of senescent cell formation increases, while their elimination is impaired by pro-survival signaling and dysregulated immune responses linked to cell death, resulting in the accumulation of senescent cells in organs [13]. Moreover, senescent cells promote tissue degeneration, aging, systemic chronic inflammation, and disease through the spread of senescence to neighboring non-senescent cells via the secretion of a variety of inflammatory cytokines, chemokines, and proteolytic factors collectively known as the senescence-associated secretory phenotype (SASP) [14]. Recent quantitative proteomics studies further demonstrate that SASP composition is highly heterogeneous and context-dependent, with disease- and tissue-specific proteoform signatures that cannot be reliably inferred from transcriptomic profiles alone [15]. Importantly, aging is closely associated with a progressive decline in autophagy and lysosomal function, which contributes to impaired cellular proteostasis and the accumulation of damaged proteins and organelles. This dysfunction is a key driver of cellular senescence, as reduced autophagic flux promotes mitochondrial dysfunction, persistent oxidative stress, and chronic activation of stress signaling pathways. In this context, selective autophagy pathways play a critical role in maintaining cellular homeostasis during aging, and their impairment reinforces multiple hallmarks of senescence, including metabolic dysregulation and inflammatory response [16,17].

### 2.2. Aging-Related Signaling Pathway

#### 2.2.1. DNA Damage and Cellular Senescence

Among various stressors capable of inducing cellular senescence, nuclear DNA damage is recognized as a fundamental senescence driver. This damage manifests as double-strand breaks (DSBs), which trigger the activation of the DNA Damage Response (DDR) pathway. The DDR functions to block cell cycle progression, thereby preventing the transcription of damaged genetic information. Inhibition of Ataxia Telangiectasia Mutated (ATM), Ataxia Telangiectasia and Rad3-related (ATR), Checkpoint Kinase 1 (CHK1), and Checkpoint Kinase 2 (CHK2), which act as mediators of DDR kinase signaling, promotes re-entry of senescent cells into the cell cycle [18,19]. Subsequently, the tumor suppressor p53 is activated and induces the expression of the cyclin-dependent kinase (CDK) inhibitor p21, which is essential for senescence-associated cell cycle arrest. p16, an inhibitor of CDK4 and CDK16, is thought to be activated during the progression of senescence to maintain the senescent phenotype. In addition, the tumor suppressor Alternate Reading Frame (ARF) has been reported to induce senescence by stabilizing p53 [20]. Proteomics-based investigations indicate that persistent DDR signaling in senescent cells is maintained not solely by sustained transcriptional activation but by altered protein turnover, stabilization of DDR-associated proteoforms, and prolonged retention of DDR complexes at sites of damage. Previous studies have reported a finely tuned regulatory system in human cancer model cells in which ATM suppresses ARF levels, while inactivation of ATM unmasks the tumor-suppressive effects of ARF [21]. In addition to stable cell cycle arrest, senescent cells acquire resistance to apoptosis through activation of pro-survival signaling pathways, including BCL-2 family proteins and PI3K/AKT signaling. This apoptosis-resistant phenotype enables the persistence of senescent hepatocytes despite sustained cellular damage and contributes to their accumulation in liver disease [22,23].

#### 2.2.2. Telomere Dysfunction and Cellular Senescence

One of the earliest discovered and best-characterized mechanisms of cellular senescence is the shortening of chromosomal telomeres. Critically shortened or dysfunctional telomeres activate ATM/ATR-dependent DDR pathways, leading to p53-p21-mediated cell cycle arrest and stabilization of the senescent phenotype [18]. Importantly, persistent DDR signaling can also occur independently of telomere length through chronic oxidative stress and mitochondrial dysfunction [24].

In liver disease, hepatocytes exposed to chronic metabolic overload, lipotoxicity, and oxidative stress exhibit sustained DDR activation, which contributes to hepatocyte senescence and progression of MASLD toward MASH. Persistent telomere-associated DDR signaling in senescent hepatocytes is closely linked to mitochondrial dysfunction, inflammatory signaling, and defective organelle quality control. Recent proteomics-based studies further suggest that chronic DDR activation in steatotic hepatocytes is associated with remodeling of mitochondrial, nuclear, and stress-response proteomes, reinforcing senescence-associated functional decline and inflammatory microenvironmental remodeling [25,26,27].

#### 2.2.3. Oncogenes and Cellular Senescence

Activation of oncogenes promotes cellular senescence. Persistent oncogenic and stress-responsive signaling can induce DNA replication stress and sustained DNA damage response (DDR) activation, thereby promoting oncogene-induced senescence (OIS) [28]. In addition, oncogenic stress enhances reactive oxygen species (ROS) production and mitochondrial dysfunction, which reinforce senescence-associated growth arrest and inflammatory signaling [29].

In liver disease, chronic metabolic stress and lipotoxicity can cooperate with oncogenic and stress-responsive pathways to promote hepatocyte senescence and progression of MASLD toward MASH. Persistent oxidative stress and mitochondrial dysfunction in steatotic hepatocytes further amplify DDR signaling and inflammatory microenvironmental remodeling, thereby increasing susceptibility to hepatocellular carcinoma (HCC). Recent proteomics-based studies additionally suggest that senescent hepatocytes undergo coordinated remodeling of mitochondrial, metabolic, and stress-response protein networks associated with oncogenic stress adaptation and disease progression [30,31].

#### 2.2.4. Mitochondrial Dysfunction and Cellular Senescence

The escalation of oxidative stress in senescent cells is intrinsically linked to the accumulation of dysfunctional mitochondria [7]. A defining feature of senescent hepatocytes is impaired mitochondrial quality control, characterized by reduced mitochondrial membrane potential (MMP), defective mitochondrial respiration, and impaired mitophagy [26]. Mitochondrial dysfunction promotes excessive reactive oxygen species (ROS) generation, which sustains persistent DNA damage response (DDR) signaling and reinforces hepatocyte senescence [32]. In senescent cells, accumulation of cytosolic DNA derived from genomic instability or mitochondrial damage activates the cGAS-STING-NF-kB signaling axis, which drives innate immune responses and promotes SASP production. This pathway serves as a critical link between DNA damage, inflammatory signaling, and senescence-associated phenotypes [33,34]. Proteomic profiling of senescent cells consistently demonstrates broad reorganization of mitochondrial proteomes, including reduced abundance of respiratory chain components, altered mitophagy-related proteins, and accumulation of damaged mitochondrial proteoforms [35]. In MASLD/MASH, chronic lipid overload and defective mitophagy further exacerbate mitochondrial proteome remodeling and oxidative stress, thereby promoting inflammatory signaling, hepatocyte senescence, and disease progression. Recent proteomics-based studies additionally suggest that dysfunctional mitochondria in steatotic hepatocytes accumulate stress-associated proteoforms and altered metabolic enzymes, contributing to persistent organelle dysfunction and inflammatory microenvironmental remodeling [36,37].

#### 2.2.5. Non-Cell-Autonomous Regulation of Senescence and Aging

Cellular senescence is increasingly recognized as a non-cell-autonomous process regulated not only by intracellular stress responses but also by intercellular communication within the tissue microenvironment. Senescent cells actively influence neighboring cells through the senescence-associated secretory phenotype (SASP), which consists of inflammatory cytokines, chemokines, growth factors, and matrix-remodeling proteins. Persistent SASP signaling can induce paracrine senescence in surrounding cells, amplify chronic inflammation, and promote tissue dysfunction during aging [38].

In the liver, senescent hepatocytes communicate extensively with non-parenchymal cells, including hepatic stellate cells (HSCs), Kupffer cells, liver sinusoidal endothelial cells (LSECs), and infiltrating immune cells. Through secretion of IL-6, IL-1β, TNF-α, TGF-β, and other SASP-associated mediators, senescent hepatocytes promote inflammatory signaling, fibrogenic activation, immune remodeling, and progression of MASLD toward MASH. In addition, accumulation of cytosolic DNA and mitochondrial damage in senescent hepatocytes can activate the cGAS-STING-NF-kB signaling axis, further reinforcing inflammatory microenvironmental remodeling and senescence propagation [39,40].

Recent proteomics-based studies further suggest that senescence-associated intercellular communication is regulated at the level of secreted protein composition and proteoform remodeling, highlighting the importance of proteomics approaches for understanding aging-associated tissue crosstalk and liver disease progression [41].

### 2.3. Biomarkers of Senescent Cells

**Figure 2:**
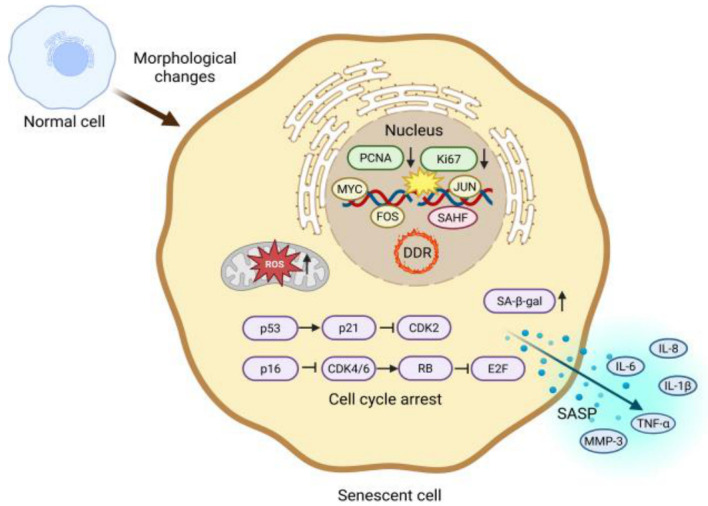
**Biomarker associated with cellular senescence.** Senescent cells exhibit enlarged, flattened morphology and a disproportionate increase in the cytoplasm-to-nucleus ratio. Typically, it is manifested by an increase in the expression of SA-β-gal and a decrease in the expression of proliferation markers such as PCNA and Ki-67, and senescent cells can also be identified by an increased expression of p21 and p16 in the cell. p53 is activated by telomere attrition and intranuclear DNA damage caused by oncogene activation, resulting in increased p21 expression. p16 expression is also presumed to be induced, and these CDK inhibitors activate RB and inhibit E2F activity, thereby causing cell cycle arrest, which serves as an indicator of senescent cells. In addition, SAHF is associated with nuclear aging and components of the Senescence-Associated Secretory Phenotype (SASP), which are components of inflammatory cytokines such as IL-6, IL-8, IL-1β, TNF-α, and the EMC degrading enzyme MMP-3 are biomarkers associated with cellular senescence. SA-β-gal; Senescence-Associated β-galactosidase, PCNA; Proliferating Cell Nuclear Antigen, Ki67; Kiel 67 antigen, SAHF; Senescence-Associated Heterochromatin Foci, CDK2; Cyclin-Dependent Kinase 2, CDK4/6; Cyclin-Dependent Kinase 4/6, RB; Retinoblastoma protein, E2F; E2 promoter-binding factor, SASP; Senescence-Associated Secretory Phenotype, IL-6; Interleukin-6, IL-8; Interleukin-8, IL-1β; Interleukin-1 beta, TNF-α; Tumor Necrosis Factor-alpha, MMP-3; Matrix Metalloproteinase-3.

The first and most widely utilized biomarker for detecting senescent cells in both cultured cells and freshly excised tissue samples is the accumulation of the lysosomal enzyme Senescence-Associated *β*-galactosidase (SA-*β*-gal) [42]. This marker can be detected via histochemical staining in most senescent cells but is generally absent in pre-senescent, quiescent, or transformed cells. Proteomics-based analyses suggest that increased SA-*β*-gal activity reflects broader lysosomal proteome expansion and altered lysosomal enzyme composition in senescent cells [43] (Figure 2).

Another hallmark of senescent cells is their aberrant morphological transformation, characterized by an enlarged and flattened shape with a disproportionately increased cytoplasmic-to-nuclear ratio. Recent studies suggest that this increase in cell size may play a causal role in inducing senescence-associated growth arrest [44]. Furthermore, in vivo studies have shown that SA-*β*-gal-positive senescent cells exhibit significantly larger sizes compared to SA-*β*-gal-negative populations [45].

A definitive marker for senescence is the loss of DNA replication capacity. This is typically assessed by immunostaining for incorporated nucleoside analogs or proliferation markers such as Ki-67 (Kiel 67 antigen) and PCNA (Proliferating Cell Nuclear Antigen), whose expression is characteristically reduced in senescent cells. However, it should be noted that some markers may not clearly distinguish senescent cells from those in a quiescent or post-mitotic state.

The cyclin-dependent kinase (CDK) inhibitors p21 and p16 are components of the tumor suppressor pathways regulated by p53 and RB, respectively, and they accumulate significantly in senescent cells. Cellular stresses such as telomere shortening and oncogene activation induce DNA damage, activate p53, and subsequently upregulate p21 expression. Persistent DNA damage is also thought to drive the expression of p16. These CDK inhibitors activate RB and suppress E2F (E2 promoter-binding factor) activity, thereby inducing cell cycle arrest [46]. Proteoform-specific regulation of p53, p21, and p16-including phosphorylation and stability differences, has been reported in senescent contexts, underscoring the importance of proteoform level analysis.

Finally, inflammatory cytokines such as IL-6 (Interleukin-6) and IL-8 (Interleukin-8), IL-1β (Interleukin-1 beta), TNF-α (Tumor Necrosis Factor-alpha), and MMP-3 (Matrix Metalloproteinase-3), which are components of Senescence-Associated Secretory Phenotype (SASP), are indicators used to evaluate general tissue or cell culture senescence at the transcriptome and protein levels. However, SASP alone cannot serve as a reliable senescence marker, as certain senescence programs lack robust SASP induction [47]. Thus, integrated transcriptomic and proteomic approaches, particularly those capturing proteoform heterogeneity, are essential for defining robust and context-specific biomarkers of cellular senescence and for guiding senescence-targeted therapeutic strategies. Importantly, in liver diseases such as MASLD, hepatocyte senescence is driven by the convergence of multiple stress-responsive pathways at the molecular level. Persistent DNA damage response (DDR) signaling, mitochondrial dysfunction-associated reactive oxygen species (ROS) production, impaired selective autophagy, and dysregulated iron metabolism collectively promote the induction and stabilization of the senescent phenotype. Proteomics-based studies further reveal that these processes are accompanied by coordinated remodeling of hepatocyte proteomes, including alterations in metabolic enzymes, mitochondrial proteins, and stress-response networks. These molecular changes not only reinforce cell-autonomous senescence but also contribute to disease progression through paracrine signaling and microenvironmental remodeling [48].

## 3. Cell Death Pathways in Hepatocyte Senescence and MASLD/MASH Progression

**Figure 3:**
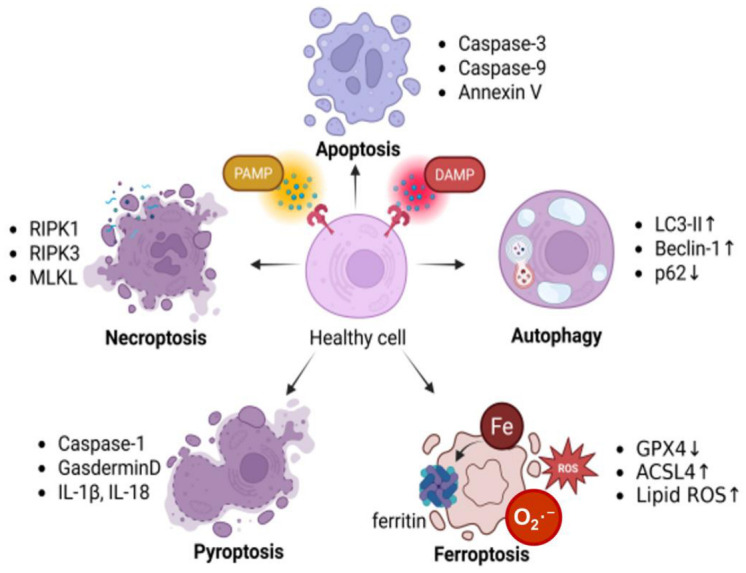
**Types of Cell Death and Their Key Markers.** When damage signals such as PAMPs or DAMPs, released due to infection, inflammation, or tissue injury, are detected from the extracellular environment, cell death is triggered as a defensive response. In necroptosis, RIPK1, RIPK3, and MLKL are expressed. Apoptosis is characterized by the expression of Caspase-3, Caspase-9, and Annexin V. Autophagy increases in LC3-II and Beclin-1 levels, accompanied by a decrease in p62. Ferroptosis (iron-dependent cell death) features a decrease in GPX4 and increases in ACSL4 and lipid ROS. Lastly, pyroptosis is marked by the expression of Caspase-1, Gasdermin D, IL-1β, and IL-18. PAMP; Pathogen-Associated Molecular Pattern, DAMP; Damage-Associated Molecular Pattern, RIPK1; Receptor-Interacting Protein Kinase, RIPK3; Receptor-Interacting Protein Kinase 3, MLKL; Mixed Lineage Kinase Domain-Like protein, GPX4; Glutathione Peroxidase 4, ACSL4; Acyl-CoA Synthetase Long Chain Family Member 4, LC3-II; Microtubule-Associated Protein 1 Light Chain 3, form II.

Regulated cell death pathways are critically involved in hepatocyte injury, inflammatory signaling, and tissue remodeling during MASLD/MASH progression. Chronic metabolic overload, mitochondrial dysfunction, oxidative stress, and defective organelle quality control activate multiple forms of hepatocyte death, which contribute to senescence-associated liver dysfunction and inflammatory microenvironmental remodeling. However, when large numbers of cells die simultaneously and accumulate as a result of infection, inflammation, or tissue injury in the extracellular environment. These released molecules are sensed as damage signals in the form of pathogen-associated molecular patterns (PAMPs) or damage-associated molecular patterns (DAMPs), thereby initiating host defense mechanisms, including inflammatory and antiviral responses [49]. From a proteomics perspective, distinct modes of cell death are increasingly defined by characteristic protein abundance patterns, post-translational modifications, and organelle-specific proteome remodeling rather than by morphology alone [50]. This review focuses on the major forms of cell death—Necrosis, Apoptosis, and Autophagy—and their links to the mechanisms of cellular senescence (Figure 3).

In MASLD/MASH, excessive lipotoxicity and mitochondrial dysfunction promote necrotic and necroptotic hepatocyte death, resulting in the release of DAMPs and amplification of inflammatory signaling within the liver microenvironment [51,52]. Proteomics studies of necrotic and necroptotic contexts reveal enrichment of damage-associated protein signatures, stress-induced proteoforms, and disrupted cytoskeletal and membrane-associated protein networks, reflecting catastrophic loss of cellular organization.

In steatotic hepatocytes, apoptosis is induced by lipotoxic stress, ER stress, mitochondrial dysfunction, and chronic inflammatory signaling, thereby contributing to hepatocyte loss and fibrosis progression during MASLD/MASH [53,54]. At the proteome level, apoptosis is characterized by caspase-mediated cleavage of specific substrates, generation of distinct cleavage-derived proteoforms, and coordinated loss of mitochondrial and nuclear protein integrity-features that are readily captured by mass spectrometry-based proteomics but are poorly resolved by transcriptomic analyses alone [55,56]. Thus, apoptosis represents a paradigm in which proteoform-specific information is essential for accurate pathway identification [57].

Autophagy is primarily a cytoprotective process that maintains cellular homeostasis by degrading and recycling damaged proteins and organelles. Although autophagy has been referred to as a form of type II programmed cell death, it is not inherently a cell death mechanism. Instead, under conditions of excessive or dysregulated stress, autophagy can contribute to a caspase-independent form of regulated cell death, often termed type II cell death [58]. Selective autophagy pathways are essential for maintaining hepatocyte proteostasis and organelle quality control during metabolic stress. In MASLD/MASH, impaired autophagic flux and lysosomal dysfunction promote the accumulation of damaged mitochondria, lipid droplets, and oxidized proteins, thereby reinforcing hepatocyte senescence and inflammatory signaling [59,60,61]. Proteomics has been instrumental in distinguishing productive autophagic flux from autophagy-associated cell death by revealing static accumulation of autophagy-related proteins, altered lysosomal proteomes, and impaired cargo degradation signatures that cannot be inferred from LC3-based assays alone [62,63].

Ferroptosis is a form of unregulated cell death characterized by excessive lipid peroxides (LPO). Ferroptosis is a distinct, iron-dependent form of regulated cell death characterized by excessive lipid peroxidation [64]. It is morphologically, biochemically, and genetically distinct from apoptosis, necrosis, and autophagy. Hallmarks include loss of plasma membrane integrity, mitochondrial shrinkage, increased membrane density, reduced or absent cristae, and outer mitochondrial membrane rupture. Ferroptosis is closely linked to dysregulated iron metabolism and oxidative stress.

Proteomics-based studies have defined ferroptosis by coordinated alterations in iron-handling proteins, antioxidant enzymes, lipid metabolism–associated proteins, and ferroptosis-specific proteoform changes (e.g., GPX4 depletion and ACSL4 enrichment), providing molecular signatures that distinguish ferroptosis from other cell death modalities [65,66].

Iron homeostasis is regulated by ferritin, composed of ferritin heavy (FTH1) and light (FTL) chains, and by NCOA4-mediated ferritinophagy, which releases intracellular iron under specific conditions [67]. Dysregulated ferritinophagy increases labile Fe^2+^ pools, enhances reactive oxygen species (ROS) generation via Fenton chemistry, and drives lipid peroxidation, ultimately promoting ferroptotic cell death.

Pyroptosis is an inflammatory form of programmed cell death induced by activation of inflammasome sensors, including members of the NOD-like receptor (NLR) family, AIM2, and Pyrin receptors [49]. Inflammasomes detect PAMPs and DAMPs released during infection or cellular stress, leading to activation of caspase-1, cleavage of pro-IL-1β and pro-IL-18, and formation of Gasdermin D (GSDMD) pores that disrupt plasma membrane integrity. Proteomic analyses of pyroptotic cells reveal characteristic enrichment of inflammasome components, processed cytokine proteoforms, and pore-forming gasdermin fragments, enabling discrimination of pyroptosis from other lytic death pathways at the protein level [68]. According to the Nomenclature Committee on Cell Death (NCCD), autophagic cell death is defined as a regulated form of cell death that depends on the autophagic machinery and can be prevented by genetic or pharmacological inhibition of autophagy [69]. Therefore, elucidating the precise role of autophagy in cell death requires approaches that can resolve dynamic protein turnover, cargo selectivity, and lysosomal competence. Proteomics provides a critical framework for this distinction by capturing pathway-specific protein signatures and proteoform dynamics across different cell death modalities, thereby linking cell death mechanisms to hepatocyte senescence and liver disease pathogenesis. In the following sections, we focus on selective autophagy pathways in hepatocytes—lipophagy, mitophagy, ferritinophagy, ER-phagy, and pexophagy—and discuss how proteomics-based studies have advanced our understanding of their roles in MASLD and aging.

## 4. Subtypes and Key Biomarkers of Selective Autophagy in Hepatocytes

### 4.1. Lipophagy

Lipophagy, a type of selective autophagy, is a mechanism of lipid metabolism and plays a role in resistance to cell death. When lipophagy becomes dysfunctional, lipids can exert lipotoxicity, eventually triggering cellular demise. For instance, in Nerve Growth Factor–Differentiated PC12 (NGFDPC12) cells, docosahexaenoic acid (DHA, 22:6 n-3) enhances autophagy by upregulating ATG7 (Autophagy Related 7) and ATG12 (Autophagy Related 12). This enhancement suppresses necrosis and apoptosis, thereby protecting cells against palmitic acid (PAM)-mediated lipotoxicity (PAM-LTx) [70]. Proteomics-based analyses indicate that lipophagy is accompanied by coordinated remodeling of lipid droplet-associated proteins, autophagy machinery components, and mitochondrial metabolic enzymes, underscoring that lipophagy is regulated at the protein network level rather than by single-gene effects [71,72,73]. Lipophagy exerts cytoprotective effects by catabolizing lipids to maintain the energy supply, preventing necrosis, apoptosis, and ATP depletion caused by mitochondrial dysfunction, and facilitating the metabolism of lipotoxic molecules [74]. Excessive lipid accumulation in organs can lead to cell death due to the toxicity of saturated free fatty acids (FFAs), a phenomenon observed in various pathological states such as obesity and metabolic diseases. FFAs induce oxidative stress and are associated with the abnormal increase of hepatic lipids in non-alcoholic fatty liver disease (NAFLD) and alcoholic liver disease (ALD), which are representative liver disorders [75]. Mass spectrometry-based proteomics has enabled the identification of disease-stage-specific lipophagy-associated protein signatures in steatotic livers, revealing heterogeneity that cannot be resolved by transcriptomic profiling alone. Autophagy activity is elevated in alcoholic mouse livers and ethanol-treated hepatocytes, whereas autophagy associated with long-lived proteins does not increase [76]. These observations suggest that the selective activation of lipophagy may represent a protective response against ethanol-induced liver injury. Proteomic profiling further reveals that pharmacological modulation of autophagy alters lipid droplet proteomes and lysosomal enzyme abundance, highlighting the importance of quantitative proteomics in distinguishing adaptive lipophagy from maladaptive autophagy-associated cell death [77].

### 4.2. Mitophagy

The degradation of mitochondria via selective autophagy, known as mitophagy, is a conserved fundamental mechanism from yeast to humans and represents a key process in mitochondrial quality control. Damaged mitochondria are sequestered into autophagosomes through ubiquitin-dependent or ubiquitin-independent receptor-mediated pathways, after which they are transported to lysosomes for degradation and recycling. During oxidative phosphorylation, mitochondria continuously generate reactive oxygen species (ROS). The accumulation of ROS can lead to oxidative stress, subsequently resulting in mitochondrial damage [78]. Therefore, the removal of damaged mitochondria through mitophagy plays a pivotal role in various physiological processes, including early embryonic development, cellular differentiation, inflammation, and apoptosis [79]. Proteomics-based studies have demonstrated that mitophagy is associated with extensive remodeling of the mitochondrial proteome, including selective depletion of respiratory chain components and enrichment of mitophagy-related adaptors. Copper (Cu) exposure induces mitophagy in hepatocytes, as evidenced by an increased number of mitophagosomes and the upregulation of PINK1, Parkin, and p62 at both mRNA and protein levels. Pharmacological inhibition of autophagy exacerbates Cu-induced cell cycle arrest and apoptosis, whereas rapamycin exerts protective effects [80]. Importantly, proteomic analyses reveal that mitophagy selectively removes damaged mitochondrial proteoforms rather than uniformly reducing mitochondrial protein abundance, emphasizing the value of proteoform-level resolution in assessing mitochondrial quality control.

### 4.3. Ferritinophagy

Recent research has revealed the mechanism by which iron is released from ferritin through a process known as ferritinophagy [81]. This process requires the selective receptor NCOA4, which binds to ferritin, transports it to autophagosomes, and subsequently delivers it to ribosomes to promote ferritin degradation and iron release. This process is precisely regulated by iron levels, which can control NCOA4 stability and iron release. When intracellular iron levels are elevated, the iron-binding protein NCOA4 interacts with the ubiquitin E3 ligase HERC2 (HECT Domain and RCC1-like Domain Containing Protein 2), targeting NCOA4 for proteasomal degradation and thereby reducing ferritinophagy. In contrast, low iron levels inhibit NCOA4-HERC2 interaction, stabilizing NCOA4, increasing ferritinophagy influx, and releasing iron from lysosomes [82]. Proteomics has been instrumental in defining ferritinophagy by enabling quantitative assessment of ferritin subunits, NCOA4 abundance, and iron-regulatory protein networks under varying metabolic conditions. NCOA4-mediated ferritinophagy is a key regulator of ferroptosis, linking selective autophagy to iron-dependent lipid peroxidation [67]. In aged fibroblasts, lysosomal dysfunction impairs ferritinophagy and attenuates ferroptosis [83], whereas NCOA4 overexpression enhances ferroptotic sensitivity by increasing ferritin degradation and iron release [84]. These findings highlight ferritinophagy as a proteomics-defined pathway in which alterations in protein stability and turnover, rather than transcriptional changes, dictate ferroptotic vulnerability.

### 4.4. ER-Phagy

Endoplasmic reticulum-selective autophagy (ER-phagy) is defined as a form of selective autophagy that enables lysosomal degradation of specific ER components. It is important to distinguish ER-phagy, which targets specific misfolded or aggregation-prone proteins for degradation. ER-phagy involves the sequestration and lysosomal degradation of entire ER subdomains. This process is often triggered by the local accumulation of protein aggregates within defined ER regions, leading to receptor-mediated engulfment of ER fragments. Classical studies have demonstrated that aggregation-prone proteins such as α1-antitrypsin Z (ATZ) and misfolded pro-insulin can accumulate within ER subdomains and promote ER-phagy-mediated clearance, highlighting a key mechanistic distinction between protein-level and organelle-level quality control [85,86,87]. ER-phagy is activated under basal, non-stress conditions to maintain ER size [88]. ER-phagy is induced by calcium imbalance, accumulation of misfolded proteins, and redox perturbations. Proteomics-based studies reveal that ER-phagy selectively targets ER-resident chaperones, folding enzymes, and membrane proteins, allowing precise remodeling of the ER proteome during stress adaptation. The unfolded protein response (UPR) detects ER stress and promotes chaperone synthesis, lipid biosynthesis, and ER expansion to alleviate protein overload [89]. Concurrently, UPR signaling induces expression of autophagy-related genes and ER-phagy receptors, facilitating clearance of damaged ER components [90]. Quantitative proteomics has enabled discrimination between adaptive ER-phagy and pathological ER turnover by revealing context-dependent changes in ER proteoform composition.

### 4.5. Pexophagy

**Figure 4:**
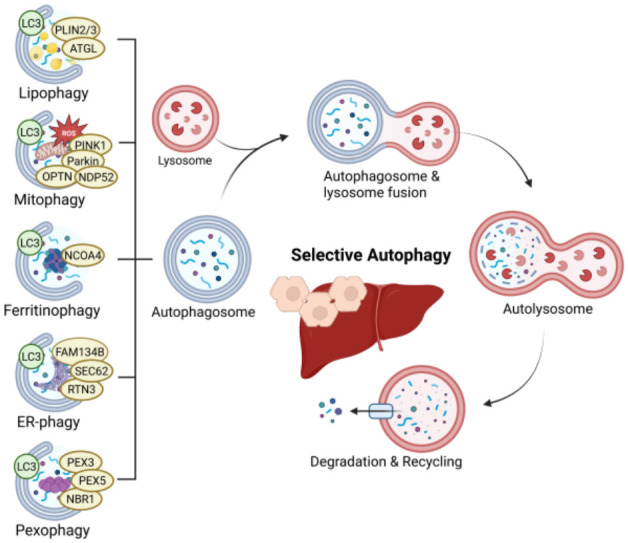
**Form, major markers, and process of selective autophagy in hepatocytes.** Among the selective autophagy forms in hepatocytes, lipophagy is decreased PLIN2/3 and increased ATGL, which promote lipophagy. PINK1, Parkin, LC3-II, OPTN, and NDP52 expression is increased in Mitophagy. NCOA4 regulates homostasis in ferritinophagy. ER-phagy increases the activity of FAM134B, SEC62, and RTN3. Pexophagy is induced by increased levels of representative proteins such as PEX3, PEX5, NBR1, and LC3-II during selective autophagy. PINK1 (PTEN-Induced Kinase 1), LC3-II; Microtubule-Associated Protein 1 Light Chain 3, form II, OPTN; Optineurin, NDP52; Nuclear Dot Protein 52 kDa, NCOA4; Nuclear Receptor Coactivator 4, FAM134B; Family with Sequence Similarity 134 Member B, SEC62; Translocation Protein SEC62, RTN3; Reticulon-3, PEX3; Peroxisomal Biogenesis Factor 3, PEX5; Peroxisomal Biogenesis Factor 5, NBR1; Neighbor of BRCA1 Gene 1.

Peroxisomes are single-membrane organelles that exist in nearly all eukaryotic cells. In mammalian cells, peroxisomes play essential roles in various cellular metabolism, including β-oxidation of fatty acids, maintaining redox homeostasis, and the biosynthesis of bile acids and plasmalogens [91]. Peroxisomes serve as central signaling hubs regulating redox and innate immunity signaling [92]. Oxidative stress disrupts peroxisomal function and induces selective autophagic degradation of peroxisomes, termed pexophagy. Proteomic analyses demonstrate that pexophagy is associated with selective loss of peroxisomal enzymes, including catalase, and remodeling of redox-regulatory protein networks. Catalase inhibition increases ROS levels and induces NBR1-dependent pexophagy under nutrient-deprived conditions [93], a phenomenon also observed in catalase knockout mice subjected to prolonged starvation [94]. Additional stressors, such as hydrogen peroxide exposure, metal chelation, or loss of heat shock proteins, further promote pexophagy. Recent proteomics-driven studies suggest that ATM-dependent ubiquitination of PEX5 triggers selective pexophagy, illustrating how post-translational modification-specific proteoforms govern peroxisome turnover. Thus, pexophagy plays a fundamental role in maintaining peroxide homeostasis, and its dysregulation contributes to the progression of metabolic and age-related diseases [95] (Figure 4).

## 5. Selective Autophagy Dysfunction in MASLD-to-MASH Progression and Hepatocyte Senescence

**Figure 5:**
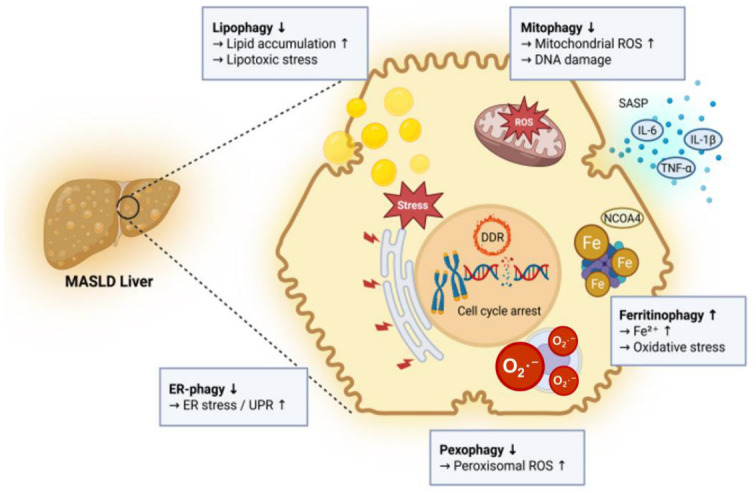
**Selective Autophagy Dysfunction Drives Hepatocyte Senescence and MASLD-to-MASH Progression.** Lipophagy dysfunction can trigger the onset of various diseases, which include non-alcoholic fatty liver disease and metabolic syndrome, by increasing the amount of lipid droplets, lipolysosomes, and autophagosomes. Mitophagy dysfunction can lead to increased reactive oxygen species (ROS) formation, mitochondrial DNA damage, and impaired mitophagy function, potentially causing hepatic steatosis, inflammation, and fibrosis. Chronic ferritinophagy in MASLD may exacerbate liver damage through iron overload and oxidative stress. Endoplasmic reticulum (ER) stress and the associated unfolded protein response (UPR) appear to contribute to NAFLD pathogenesis due to the liver’s high ER content. Finally, hepatocytes maintain peroxisome homeostasis through pexophagy, demonstrating the role of selective autophagy in the development of various liver diseases. ROS: Reactive Oxygen Species, DDR; DNA Damage Response, NCOA4; Nuclear Receptor Coactivator 4, UPR; Unfolded Protein Response, IL-6; Interleukin-6, IL-1β; Interleukin-1 beta, TNF-α; Tumor Necrosis Factor-alpha.

Importantly, these selectively autophagy pathways are closely linked to the regulation of hepatocyte senescence in liver disease. At the molecular level, impaired autophagy contributes to the accumulation of damaged organelles, persistent oxidative stress, and activation of DNA damage response (DDR) signaling, thereby promoting the induction and maintenance of the senescent phenotype in MASLD. Proteomics studies further indicate that these processes are associated with coordinated remodeling of hepatocyte proteomes, including mitochondrial dysfunction-related proteins, iron-handling proteins, and stress-response networks, which reinforce senescence-associated functional decline (Figure 5). These processes are further exacerbated during aging, where a decline in autophagy and lysosomal function contributes to hepatocyte senescence and disease progression. Collectively, representative proteomics-based alterations discussed in this section are summarized in Table 1, which provides a comprehensive overview of studies across different selective autophagy pathways in liver diseases [23,96].

### 5.1. Lipophagy

Lipophagy is a type of selective autophagy that removes unnecessary lipid droplets (LDs) by sending their contents to lysosomes for degradation by autophagosomes. Unlike adipose tissue, the liver is considered a lipid metabolic organ, and lipophagy is essential for preventing LD accumulation and maintaining lipid homeostasis [97]. Therefore, it plays a crucial role in preventing metabolic liver diseases, including non-alcoholic fatty liver disease (NAFLD) and alcoholic fatty liver disease (AFLD), and its purpose is to treat these functional disorders potentially [98]. Proteomics-based studies of MASLD livers have revealed that impaired lipophagy is associated with disease stage-dependent remodeling of lipid droplet-associated proteins, lysosomal enzymes, and autophagy machinery components, highlighting heterogeneity that cannot be captured by transcriptomic analyses alone. Pharmacological activation or restoration of autophagy promotes lipid droplet clearance, reduces pro-inflammatory mediator production, and suppresses activated hepatic stellate cells (HSCs), thereby contributing to liver function recovery and fibrosis attenuation [99]. Although no definitive cure exists, autophagy induction has been proposed as a promising therapeutic strategy [100]. Quantitative proteomics further suggests that therapeutic autophagy activation does not uniformly enhance lipophagy but selectively modulates lipid-handling proteomes, underscoring the need for protein-level stratification of MASLD. Histomorphological and molecular analyses demonstrate lipid phagocytosis dysfunction in experimental NAFLD models and patient cohorts. Accumulation of p62/SQSTM1 and LC3-II correlates with NAFLD activity score (NAS) and fibrosis stage [101]. NAFLD patients exhibit increased lipid droplets, lipolysosomes, and autophagosomes compared to healthy individuals. Proteomics-based evaluation indicates that accumulation of autophagy markers reflects stalled autophagic flux rather than enhanced degradation, emphasizing a limitation of static marker-based assessments in MASLD. Thus, impaired lipophagy sensitizes hepatocytes to apoptotic stimuli and contributes to NAFLD and metabolic syndrome progression [74]. At the molecular level, lipophagy is regulated by coordinated interactions between lipid droplet-associated proteins (e.g., PLIN family), autophagy machinery (LC3, ATG proteins), and lysosomal enzymes. Proteomics analyses further reveal that lipophagy in MASLD is associated with selective remodeling of lipid droplet proteomes and metabolic enzymes, rather than uniform lipid degradation, highlighting its role in controlling lipotoxicity and hepatocyte stress responses [102,103].

### 5.2. Mitophagy

Mitophagy is a mitochondria-specific autophagy pathway that selectively segregates and degrades damaged mitochondria to maintain functional integrity and cellular homeostasis. The most well-established mitophagy pathway is the PTEN-Induced Kinase1 (PINK1)-Parkin-dependent pathway [104]. In addition to the canonical PINK1-Parkin-dependent mechanisms. These non-canonical pathways are typically mediated by mitochondrial outer membrane receptors, including BNIP3, NIX (BNIP3L), and FUNDC1, which directly interact with LC3 to facilitate autophagosome formation. Notably, these receptor-mediated pathways are often activated under hypoxic or metabolic stress conditions and do not require ubiquitination of mitochondrial proteins. Recent proteomics studies further suggest that these alternative pathways contribute to the selective removal of damaged mitochondrial proteoforms in a context-dependent manner [105,106,107]. Mitophagy functions as a protective mechanism, preventing the generation of reactive oxygen species (ROS) caused by damaged mitochondria and maintaining redox balance. It stimulates the degradation of lipid droplets and the release of free fatty acids, which are transported to healthy mitochondria to increase β-oxidation and energy release [108]. Proteomics analyses in MASLD models reveal that defective mitophagy is accompanied by selective accumulation of dysfunctional mitochondrial proteoforms, rather than a uniform increase in mitochondrial content [109]. AMP-activated protein kinase (AMPK) is a key regulator of mitochondrial biogenesis and mitophagy. Chronic high-fat diet feeding increases mitophagy in hepatocytes, whereas disruption of AMPK signaling impairs mitochondrial quality control and exacerbates fatty liver phenotypes [110]. Autophagy dysfunction leads to ROS overproduction, mitochondrial DNA damage, inflammation, and fibrosis [111]. Proteomics-based profiling demonstrates that restoration of mitophagy rebalances mitochondrial proteomes and reduces senescence-associated protein signatures in hepatocytes. Mitophagy protects the liver from tissue damage and contributes to disease modulation in ALD, DILI, NAFLD, and viral hepatitis [112,113]. These findings highlight mitophagy as a proteomics-defined quality control pathway linking mitochondrial dysfunction to MASLD progression. Additional proteomics findings across liver disease models are summarized in Table 1.

### 5.3. Ferritinophagy

Ferritinophagy plays a dual role in liver disease. In liver fibrosis, ferritinophagy promotion of hepatic stellate cells (HSCs) can induce cellular senescence and reduce fibrosis progression. However, in non-alcoholic fatty liver disease (NAFLD), chronic ferritinophagy can exacerbate liver damage through iron overload and oxidative stress [114]. Iron accumulation leads to excessive production of reactive oxygen species (ROS), which promotes HSC fibrotic activation. Whereas blocking hepatocyte extracellular vesicle (EV) secretion or depleting iron within EVs restores hepatic iron homeostasis and alleviates NAFLD/NASH-associated hepatic steatosis and fibrosis [115]. Proteomics-based studies reveal that dysregulated ferritinophagy alters iron-handling proteomes and antioxidant defense networks, creating a molecular environment permissive for ferroptosis in MASLD [11,116] NCOA4 mediates selective autophagy of ferritin and regulates ferroptosis by controlling intracellular free iron levels. Inhibition of NCOA4 reduces iron-mediated apoptosis and liver damage, identifying NCOA4 as a potential therapeutic target [117]. At the molecular level, NCOA4 functions as a selective cargo receptor that delivers ferritin to autophagosomes, thereby regulating intracellular labile iron pools. Proteomic analyses indicate that dysregulated ferritinophagy alters iron-handling protein networks and antioxidant defense systems, promoting lipid peroxidation and ferroptosis, which are key drivers of liver injury and disease progression [118,119]. Proteoform-level regulation of ferritin subunits (FTH1 and FTL) has been identified by proteomics as a determinant of ferroptotic sensitivity. Elevated expression of ferroptosis-related genes, including FTH1 and FTL, correlates with prognosis in hepatocellular carcinoma patients [120]. These observations support a model in which ferritinophagy-driven proteome remodeling links MASLD progression, ferropotosis, and liver cancer risk.

### 5.4. ER-Phagy

The normal function of the ER, a major site for protein synthesis, folding, and modification within cells, is essential for maintaining cellular homeostasis. ER stress occurs when chronic environmental stresses affecting the ER, including genetic defects, chemical toxins, oxidative stress, and mycotoxins, lead to the accumulation of misfolded and unfolded proteins. To alleviate ER stress, the unfolded protein response (UPR) is triggered to regulate protein degradation. ER stress is a hallmark of chronic liver disease and is closely associated with the progression of various hepatic pathologies, including ALD, viral hepatitis, liver fibrosis, and hepatocellular carcinoma. ER stress has been identified as influencing major processes in ALF, including hepatocyte injury, inflammatory response, steatosis, and hepatic stellate cell (HSC) activation [87]. Proteomics analyses reveal that ER stress in MASLD is characterized by selective remodeling of ER-resident proteomes, rather than global protein degradation. FAM134B is an ER-phagy receptor implicated in cancer, neurodegeneration, and viral hepatitis [121]. Mechanistically, ER-phagy is mediated by ER-resident receptors such as FAM134B, SEC62, and RTN3, which interact with LC3 to facilitate the sequestration of ER subdomains. Proteomics studies further reveal that ER-phagy selectively removes misfolded ER proteoforms while preserving functional protein-folding machinery, thereby acting as a critical quality control mechanism in hepatocytes under metabolic stress conditions [87,122]. Dysregulation of FAM134B alters ER turnover and stress signaling. Quantitative proteomics has demonstrated that ER-phagy selectively depletes misfolded ER proteoforms while preserving functional folding machinery, distinguishing adaptive from pathological ER stress responses. Targeting ER stress-associated protein degradation has emerged as a therapeutic strategy in chronic liver disease [123,124]. These findings position ER-phagy as a proteomics-defined adaptive pathway that modulates MASLD pathogenesis.

### 5.5. Pexophagy

Peroxisomes are cellular organelles involved in various metabolic activities, such as purine degradation, long-chain fatty acid β-oxidation, bile acid synthesis, and phospholipid synthesis. These autonomously replicating organelles generate reactive oxygen species (ROS) as breakdown products of fatty acid β-oxidation. Cells maintain peroxisome homeostasis through pexophagy, which inhibits diseases such as peroxisome biogenesis disorders, oxidative damage, and cancer. Pexophagy is a catabolic process that involves the selective degradation of peroxisomes through autophagy. Recent studies suggest that peroxisome membrane proteins, such as PEX3 and PEX5, play a crucial role in regulating pexophagy. Pexophagy recycles damaged peroxisomes, which inhibits ROS production within peroxisomes [125]. Proteomics-based profiling reveals that MASLD is associated with selective loss of peroxisomal enzymes and altered redox-regulatory protein networks, consistent with impaired pexophagy. Fenofibrate, a peroxisome proliferator-activated receptor alpha (PPAR-α) agonist, prevents HFD-induced liver damage by activating genes involved in lipid metabolism and detoxification while suppressing inflammation and HSC activation [126,127]. Proteomics studies indicate that PPAR-α activation restores peroxisomal proteome integrity rather than merely increasing peroxisome number. PRGs (Peroxisome-Related Genes) influence lipid utilization, tumor progression, and immune resistance in hepatocellular carcinoma [128]. Collectively, proteomics-based evidence positions pexophagy as a critical regulator of metabolic flexibility and disease susceptibility in MASLD. At the molecular level, pexophagy is regulated by ubiquitination of peroxisomal membrane proteins (e.g., PEX5) and recognition by autophagy receptors such as NBR1 and p62. Proteomics analyses demonstrate selective loss of peroxisomal enzymes and remodeling of redox-related protein networks in MASLD, linking impaired pexophagy to oxidative stress and metabolic dysfunction [129,130].

**Table 1:** **Proteomics-based alterations in selective autophagy across liver diseases.** The table summarizes changes in protein abundance, key autophagy regulators, and organelle-specific proteome remodeling associated with different forms of liver disease.

Autophagy	Ref.	Contents	Target Disease
**Lipophagy**	[131]	Autophagy-related proteins: LC3 ↑, Beclin-1 ↑ Ferroptosis-associated proteins: GPX4 ↑, SLC7A11 ↑, ACSL4 ↓	Drug-induced liver injury
	[132]	Chaperone-associated proteins: HSP90A ↑ Lipid droplet–associated proteins: PLIN2 ↓	HCC
	[133]	Autophagy-related proteins: GLIPR2 ↓ Inflammasome-associated proteins: NLRP3 ↓	MASLD
	[134]	Metabolic enzymes: decreased abundance DNA replication–associated proteins: decreased abundance Wnt signaling–associated proteins: decreased abundance	HCC
	[135]	Lysosomal-associated proteins: LAMTOR1 ↓ MTORC1 pathway–associated proteins: mTOR ↓	MASLD
	[136]	Rac1-associated proteins: decreased abundance Lipid accumulation–associated proteins: decreased abundance	MASLD
	[137]	Antioxidant-related proteins: altered abundance Mitochondrial metabolism–associated proteins: altered abundance Iron-handling–associated proteins: altered abundance	Iron Overload-Induced Liver Injury
	[138]	Cholesterol efflux–associated proteins: decreased abundance Autophagy-related proteins: decreased abundance	Atherosclerosis
	[139]	Chaperone-mediated autophagy–associated proteins: increased abundance Apolipoprotein-related proteins (ApoB): decreased abundance mTORC2 pathway–associated proteins: altered abundance	MASLD
	[140]	Integrin β6–associated proteins: altered abundance Annexin A2–associated proteins: altered abundance Autophagy-related proteins: altered abundance	HCC
	[141]	AMPK-associated proteins: altered abundance Lipophagy-related proteins: altered abundance Lipid metabolism–associated enzymes: altered abundance	MASLD
**Mitophagy**	[142]	Autophagy-related proteins: LC3-II ↑, ATG7 ↑ Mitochondrial outer and inner membrane proteins: MFN1 ↓, MFN2 ↓, TOM20 ↓, HSP60 ↓, TIM23 ↓ Mitophagy-associated proteins: PINK1 ↑	HCC
	[143]	Mitophagy- and mitochondrial dynamics–associated proteins: MFN ↑, p62 ↑, decreased abundance of ubiquitinated proteins Quantitative proteomics (Park2 KO vs. Park2 KI HCC mouse model): - Macrophage-associated proteins: ARG1 ↑ - T-cell inhibitory–associated proteins: CEACAM1 ↑, LGALS9 ↑ - T-cell and NK cell–associated proteins: FGB ↓, MYO18A ↓	HCC
	[144]	Mitochondrial fission–associated proteins: DNM1L ↓, MTFR1 ↓, MTFP1 ↓ Mitophagy execution–associated proteins: CALCOCO2 ↓, OPTN ↓, PARK2 ↓, PARK7 ↓, PGAM5 ↓, PHB1 ↓	Polycystic ovary syndrome (PCOS), MASLD
	[145]	Mitophagy-related proteins: p62 (SQSTM1) ↑ under HFD compared with LFD; reduced abundance in OVX mice under HFD + exercise conditions Autophagy-related proteins: LC3-II, no significant change Ubiquitin-binding adaptor proteins (leupeptin-treated HFD mice): UBB ↑, OPTN ↑, NBR1 ↑, NDP52 ↑	MASLD
	[146]	Mitophagy markers (mKeima-based assay): increased red signal with APAP alone; increased green signal with APAP + saikosaponin D Organelle markers: reduced colocalization of TOM20 (mitochondrial marker) and LAMP1 (lysosomal marker) under APAP + saikosaponin D Autophagy- and SNARE-associated proteins (in vivo proteomics): SNAP29 ↑, VAMP8 ↑, STX17 ↑, RAB7A ↑ with APAP treatment; reduced abundance with combined APAP + saikosaponin D treatment	Acute liver failure (ALF)
	[147]	SLC7A11-associated proteins: altered abundance, accompanied by changes in mitochondrial membrane potential AMPKα1-associated proteins: altered abundance under SLC7A11 modulation Mitochondrial dynamics and mitophagy-related proteins: DRP1 ↓, p62 (SQSTM1) ↓ Autophagy initiation–associated proteins: ULK1-associated proteins, altered abundance	MASH
	[148]	MST1-associated proteins: altered abundance under palmitic acid treatment Mitophagy-related proteins: Parkin ↑ Autophagy-related proteins: reduced LC3-II/LC3-I ratio under palmitic acid treatment	MASLD
	[149]	TMX2-associated proteins: altered abundance Mitochondrial outer membrane–associated proteins: VDAC2 ↓, VDAC3 ↓ Mitophagy-related proteins: Parkin-associated proteins, altered abundance Autophagy-related proteins: LC3B-I ↓, LC3B-II ↑ Mitochondrial proteins: TOM20 ↓, COX IV ↓, TIM23 ↓	HCC
	[150]	Mitophagy-related proteins: PINK1 ↓, Parkin ↓, Beclin1 ↓, reduced LC3-II/LC3-I ratio under free fatty acid treatment ZNF143- and NEAT1-associated conditions: increased abundance of PINK1, Parkin, Beclin1, and LC3-II/LC3-I ratio upon knockdown ROCK2-associated proteins: altered abundance, modulating mitophagy-related protein profiles	MASLD
	[151]	Mitophagy-related proteins: PINK1 ↓, Parkin ↓ under alcohol exposure; increased abundance under DUSP1-overexpressing conditions Autophagosome cargo protein: p62 (SQSTM1) ↓ under alcohol exposure; restored abundance under DUSP1-overexpressing conditions Autophagy-related proteins: mitochondrial LC3-II ↓ under alcohol exposure; increased abundance under DUSP1-overexpressing conditions	Alcohol-related liver disease (ALD)
	[152]	PCSK9-associated proteins: decreased abundance under PCSK9 blockade PTEN-associated proteins: increased abundance under PCSK9 blockade Mitophagy-related proteins: PINK1 ↑, Parkin ↑	Hepatic ischemia-reperfusion injury
	[153]	Autophagy/mitophagy markers: LC3-II ↑, p62 ↓, increased LAMP1–mitochondria colocalization and mt-Keima red signal Mitochondrial proteins: decreased abundance of TOM20 and COX IV Parkin-associated changes: increased mitochondrial translocation of Parkin and ubiquitination of mitochondrial proteins Mitochondrial outer membrane proteins: MFN1/2 ↓, VDAC ↓	MASLD
	[154]	Autophagy/mitophagy-related proteins: LC3-II ↑, p62 ↓ Mitochondrial proteins: TOM20 ↓ RABIF-dependent conditions: loss of LC3-II increase and restoration of TOM20 abundance under RABIF knockdown	HCC
**Ferritinophagy**	[155]	Ferroptosis- and ferritinophagy-related proteins: PTGS2 ↓, ACSL4 ↓, GPX4 ↑, SLC7A11 ↑, FTH1 ↑, NCOA4 ↓ Oxidative stress–associated proteins: CAT ↑, SOD ↑; reduced lipid peroxidation markers (4-HNE, MDA) Mitophagy-related proteins: PINK1 ↓, Parkin ↓ under glycyrrhetinic acid treatment	DON-induced liver injury
	[156]	Ferritinophagy-related proteins: NCOA4 ↑ under LPS/iE-DAP treatment; reduced abundance with quercetin; FTH1 ↓ under LPS/iE-DAP, preserved with quercetin Ferroptosis-related proteins: GPX4 ↓ under LPS/iE-DAP; increased abundance with quercetin Iron homeostasis–associated proteins and markers: increased free iron and lipid peroxidation markers under LPS/iE-DAP; reduced levels with quercetin; iron efflux protein FPN1 ↑ with quercetin	LPS/iE-DAP-induced acute liver injury
	[157]	LACTB-associated proteins: reduced abundance in hepatocellular carcinoma tissues; increased abundance under LACTB overexpression Ferritinophagy-related proteins: HSPA8 ↓, NCOA4 ↑ under LACTB overexpression Ferroptosis-related proteins: SLC7A11 ↓, GPX4 ↓, accompanied by increased lipid peroxidation and ROS markers	HCC
	[158]	Ferritinophagy-related proteins: NCOA4 ↑, accompanied by increased ferritin degradation and intracellular iron accumulation Ferroptosis-related proteins and markers: PTGS2 ↑, increased lipid peroxidation markers (e.g., MDA), reduced antioxidant defense–associated proteins (GPX4 ↓, GSH ↓) Autophagy-related proteins: LC3B-II/I ↑, p62 ↓	Acetaminophen (APAP) and ischaemia/reperfusion (I/R)-induced liver injury
	[159]	Ferritinophagy-related proteins: NCOA4 ↑, decreased ferritin abundance, accompanied by increased intracellular free iron Modulation by iron/autophagy perturbation: attenuation of ferritinophagy- and ferroptosis-associated protein changes by chloroquine or iron chelation (DFO)	Cadmium-induced liver dysfunction
	[160]	Ferritinophagy-related proteins: NCOA4 ↑, accompanied by increased ferritin degradation Iron- and oxidative stress–associated markers: increased labile iron (Fe^2+^) and elevated reactive oxygen species Mitochondrial function–associated markers: increased mitochondrial ROS, decreased mitochondrial membrane potential, and reduced ATP levels	Acetaminophen-induced acute liver injury
	[161]	TFEB- and ferritinophagy-related proteins: TFEB-associated proteins ↑, NCOA4 ↑, accompanied by increased ferritin degradation Iron- and lipid peroxidation–associated markers: increased labile iron pool (Fe^2+^) and elevated lipid ROS levels Modulation by TFEB inhibition: reduced NCOA4-dependent ferritinophagy, decreased labile iron and oxidative stress–associated markers	Ionophore polyether antibiotics (IPAs)-induced hepatotoxicity
	[162]	Ferritinophagy-related proteins: NCOA4 ↑, accompanied by increased ferritin degradation Iron- and oxidative stress–associated markers: increased labile iron (Fe^2+^), elevated reactive oxygen species and lipid peroxidation markers Autophagy- and ferroptosis-related features: altered autophagy-associated protein profiles consistent with ferroptotic cell death–related changes	DON-induced liver injury
	[163]	Ferritinophagy-related proteins: NCOA4 ↑ under reduced HNF4A conditions; decreased abundance with HNF4A overexpression Ferroptosis- and antioxidant defense–associated proteins: GPX4 ↓, SLC7A11 ↓, accompanied by reduced GSH and increased lipid peroxidation markers (MDA, ROS) Iron homeostasis–associated markers: increased intracellular labile iron (Fe^2+^) under ferritinophagy-active conditions	HBV-related acute-on-chronic liver failure (HBV-ACLF)
	[164]	Ferritinophagy- and iron homeostasis–associated proteins: increased iron accumulation consistent with enhanced ferritin degradation under AFB1 exposure Ferroptosis- and antioxidant defense–associated proteins: SLC7A11 ↓, GPX4 ↓ under AFB1 exposure; increased abundance under 4-methylesculetin supplementation Stress- and cell death–associated protein profiles: altered abundance of ER stress– and apoptosis-related proteins under AFB1 exposure, attenuated with 4-methylesculetin	Aflatoxin B1(AFB1)-induced liver injury
**ER-phagy**	[165]	Autophagy-related proteins: LC3-II ↑, p62 ↓ Lysosomal-associated proteins: LAMP1 ↑, LAMP2 ↑ HMMR-associated protein changes: altered abundance correlated with autophagolysosomal protein profiles under ER stress conditions	HCC
	[166]	Reticulophagy-related proteins: reduced abundance of ER-resident proteins (Calnexin ↓, PDI ↓) with increased lysosomal localization ER stress–associated proteins: CHOP ↓ under FAM134B-associated conditions Autophagy/lysosome-related features: protein abundance patterns consistent with selective ER turnover	Hepatocyte apoptosis
	[167]	ER-phagy receptor–associated proteins: increased abundance under SAHA treatment Autophagy-related markers: increased LC3–ER colocalization ER stress–associated protein profiles: altered abundance consistent with enhanced ER turnover	HCC
	[168]	ER-phagy–related features: reduced ER-phagy–associated protein profiles ER stress–associated proteins: GRP78 ↑, CHOP ↑ Autophagy/ER turnover–related markers: altered abundance patterns consistent with impaired ER homeostasis	ALD
	[169]	Reticulophagy-related proteins: reduced LC3–ER association consistent with altered ER turnover ER stress–associated proteins: increased abundance of ER stress–related markers RETREG1/FAM134B-associated protein profiles: altered abundance patterns under CKAP4-associated conditions	HCC
	[87]	ER-phagy–related proteins: altered abundance patterns consistent with enhanced ER turnover under FAM134B-associated conditions ER stress–associated proteins: GRP78 ↓, CHOP ↓ Fibrosis-associated proteins: α-SMA ↓, collagen I ↓	ALF
	[170]	Reticulophagy-related proteins: RETREG1 ↑ under lipotoxic conditions ER stress–associated proteins: CHOP ↓, consistent with altered ER stress–related protein profiles ER turnover–associated features: protein abundance patterns consistent with enhanced ER-phagy–related processes	MASLD
	[171]	ERAD-associated proteins: reduced abundance of HRD1 ↓ and SEL1L ↓ ER-phagy–related proteins: increased ER-phagy–associated protein profiles ER stress/apoptosis–associated protein profiles: altered abundance patterns consistent with modified ER stress–related responses	HCC
	[172]	Reticulophagy-related proteins: increased abundance of FAM134B under AICAR-treated conditions Autophagy-related markers: increased LC3–ER association consistent with enhanced ER turnover ER stress–associated protein profiles: altered abundance patterns under lipotoxic conditions with AICAR treatment	MASLD
	[173]	Reticulophagy-related proteins: reduced abundance of FAM134B Autophagy-related markers: decreased LC3–ER colocalization ER stress–associated protein profiles: altered abundance patterns consistent with modified ER stress–related responses	Steatotic liver
**Pexophagy**	[94]	Peroxisome-associated proteins: reduced abundance of catalase, PMP70 ↓, and PEX14 ↓ Oxidative stress–associated markers: increased reactive oxygen species under catalase-deficient and fasting conditions Autophagy-related protein profiles: altered abundance patterns consistent with enhanced peroxisome turnover	Liver damage induced by prolonged starvation
	[174]	Pexophagy-related proteins: increased abundance of NBR1, p62, PEX5, PEX14, LC3, ATG7, and LAMP2 under clofibrate treatment Peroxisome-associated proteins: altered abundance patterns consistent with enhanced peroxisome turnover Oxidative stress–associated proteins: increased abundance of catalase and SOD2	MASLD
	[175]	Peroxisome-associated proteins: reduced abundance of catalase, ACOX1, MFP1, UOX, PEX14, and PEX3, consistent with decreased peroxisome content Autophagy-related proteins: increased LC3-II/LC3-I ratio and reduced p62 abundance, consistent with enhanced organelle turnover Pexophagy-associated proteins: altered abundance of NBR1 and p62 associated with peroxisome clearance	Peroxisomal disorder-like liver cirrhosis
	[176]	Hepatic stellate cells: reduced abundance of TFEB-associated autophagy- and fibrosis-related protein modules Hepatocytes: increased abundance of autophagy-associated lipid metabolism–related proteins	Liver fibrosis
	[177]	Increased abundance of mitochondrial respiratory chain and biogenesis–associated proteins (e.g., COXIV) Increased abundance of mTORC1 downstream signaling and autophagy substrate–associated proteins (p-4EBP1, p-S6, LC3-II, p62), indicating impaired autophagic flux Increased abundance of oxidative stress– and organelle damage–associated protein signatures (ubiquitinated mitochondrial proteins, 4-HNE), associated with fasting-induced liver pathology	Metabolic liver impairment in zebrafish with PEX5 deficiency

## 6. Future Therapeutic Perspectives and Clinical Implications

Growing evidence supports that hepatocyte senescence is not merely a passive consequence of aging but an active driver of MASLD progression through persistent DNA damage response (DDR) signaling, mitochondrial dysfunction, organelle stress, and chronic inflammatory cues. In this context, selective autophagy pathways—lipophagy, mitophagy, ferritinophagy, ER-phagy, and pexophagy—represent a mechanistic convergence point linking metabolic overload to senescence-associated loss of proteostasis and organelle quality control. Proteomic remodeling in hepatocytes should not be interpreted solely as a downstream consequence of cellular stress. While initial alterations in protein abundance and organelle-specific proteomes are triggered by stressors such as persistent DNA damage response (DDR), mitochondrial dysfunction, and metabolic overload, these changes can in turn reinforce and stabilize the senescent phenotype through feedback mechanisms, including impaired organelle quality control and chronic inflammatory signaling. Thus, proteome remodeling functions both as a consequence and as a driver of hepatocyte senescence, forming a self-reinforcing loop that contributes to MASLD progression. Dysfunction of selective autophagy pathways may further reinforce this apoptosis-resistant state by promoting the accumulation of damaged organelles, sustaining oxidative stress, and maintaining activation of pro-survival signaling pathways in senescent hepatocytes. Notably, selective autophagy plays a key role in modulating cGAS-STING signaling by removing cytosolic DNA and damaged mitochondria. Impaired autophagy can therefore lead to persistent activation of the cGAS-STING-NF-kB axis, amplifying inflammatory signaling and SASP, whereas intact autophagy restrains this pathway and limits chronic inflammation in senescent hepatocytes [22,178,179]. Importantly, proteomics-based studies indicate that these pathways are dysregulated at the level of protein abundance, turnover, subcellular localization, and proteoform composition, rather than through uniform transcriptional changes. Therapeutic strategies that restore or reprogram selective autophagy may therefore provide dual benefits: (i) improvement of hepatic metabolic homeostasis and (ii) attenuation of senescence burden and its non-cell-autonomous propagation via SASP.

### 6.1. Targeting Senescence Itself: Senolytics, Senomorphics, and DDR Modulators

From a translational perspective, the most straightforward strategy is to reduce the abundance or impact of senescent hepatocytes and related senescent populations in the liver microenvironment. Senolytics aim to selectively eliminate senescent cells by exploiting their dependence on pro-survival signaling networks (e.g., BCL-2 family–related survival, PI3K/AKT signaling, and other anti-apoptotic programs). In contrast, senomorphics (or senostatics) attempt to suppress SASP and senescence-associated phenotypes without killing cells, potentially reducing systemic inflammation and paracrine senescence spread [14]. While these approaches are conceptually attractive, liver-specific safety and selectivity remain critical issues. Proteomics-based profiling of senescent hepatocytes has revealed heterogeneity in survival signaling pathways, suggesting that senolytic sensitivity may depend on senescence-associated protein signatures rather than on single markers such as p16 or p21 alone.

DDR signaling itself represents another intervention node. Persistent DDR at telomeric regions can maintain senescence even when telomere shortening is not the sole driver [18,24]. Quantitative proteomics suggests that chronic DDR signaling in senescent cells is sustained by stabilization of DDR-associated proteoforms and impaired protein turnover, raising the possibility of selectively targeting maladaptive DDR outputs rather than core repair functions. Accordingly, next-generation senescence-targeting therapies should integrate tissue targeting, intermittent dosing, and proteomics-informed pharmacodynamic monitoring using senescence-associated protein and proteoform panels rather than relying solely on static histological markers.

### 6.2. Restoring Proteostasis and Nutrient Sensing to Re-Enable Autophagy

Autophagy is tightly regulated by nutrient-sensing pathways, and MASLD is characterized by nutrient excess combined with defective intracellular recycling. Canonical interventions include AMPK activation and mTORC1 inhibition, which promote catabolic and recycling programs. Proteomics-based analyses reveal that effective autophagy restoration requires coordinated recovery of lysosomal proteomes and cargo degradation capacity, not merely increased autophagosome formation. Clinically, achieving hepatocyte-specific autophagy modulation without systemic side effects remains challenging. Liver-targeted delivery strategies, combined with proteomics-guided assessment of autophagy flux and lysosomal competence, may enable more precise therapeutic calibration [42].

### 6.3. Lipophagy as a Therapeutic Lever for Hepatic Steatosis and Lipotoxicity

Given that hepatic lipid overload is a primary driver of MASLD, restoring lipophagy is an attractive strategy to reduce lipotoxic stress. However, excessive lipid mobilization may transiently increase free fatty acid flux and aggravate mitochondrial oxidative stress if mitochondrial quality control is not concurrently improved. Proteomics studies indicate that effective lipophagy enhancement is associated with coordinated remodeling of lipid droplet–associated proteomes and mitochondrial metabolic enzymes, supporting combination strategies that couple lipophagy induction with mitophagy activation. Clinical translation will require biomarkers that capture lipophagy status. Proteomics-derived lipid handling signatures and lysosome–lipid droplet interaction proteoforms may outperform conventional LC3/p62 measurements for patient stratification across MASLD stages.

### 6.4. Mitophagy and Mitochondrial Quality Control to Break the ROS–DDR–Senescence Loop

Mitochondrial dysfunction establishes a self-reinforcing loop of ROS production, DDR activation, and senescence maintenance. Proteomics-based profiling demonstrates that mitophagy selectively eliminates damaged mitochondrial proteoforms rather than globally reducing mitochondrial content, highlighting proteoform-level resolution as essential for therapeutic monitoring. Therapeutic enhancement of mitophagy may therefore interrupt the ROS–DDR–senescence axis, particularly when combined with interventions that limit lipid-induced oxidative stress.

### 6.5. Ferritinophagy–Iron Axis: Precision Modulation to Prevent Oxidative Damage and Ferroptosis Imbalance

Iron homeostasis is intimately linked to both metabolic disease and aging biology, and ferritinophagy (NCOA4-mediated ferritin turnover) is a central regulator of labile iron pools [81,82]. Proteomics reveals that ferritinophagy dysregulation alters iron-handling proteomes and antioxidant defense networks in MASLD, underscoring the need for precision modulation rather than uniform activation or inhibition. Translational development will require identification of iron-loaded versus iron-restricted phenotypes. Proteomics-based stratification of ferritin and NCOA4 proteoforms may guide patient selection and therapeutic timing while minimizing systemic hematologic risks.

### 6.6. ER-Phagy and Proteotoxic Stress Relief to Prevent Inflammation and Fibrogenesis

ER stress and unfolded protein response (UPR) activation are major features of metabolic liver disease and can drive inflammation and hepatocyte injury. ER-phagy is positioned as a quality control pathway to remove damaged ER fragments and protein aggregates, thereby restoring ER homeostasis [88]. Proteomics studies demonstrate that adaptive ER-phagy selectively clears misfolded ER proteoforms while preserving functional folding machinery, whereas maladaptive ER stress is associated with broad proteome destabilization. Therapeutic strategies should therefore aim to restore adaptive ER-phagy rather than globally suppress ER stress responses. Proteomics-informed ER stress and ER-phagy biomarkers may enable disease staging and prediction of therapeutic responsiveness.

### 6.7. Pexophagy and Peroxisome-Directed Strategies to Regulate Redox and Lipid Oxidation

Peroxisomes regulate fatty acid oxidation and ROS metabolism, positioning pexophagy as a relevant pathway in MASLD. Proteomics-based analyses reveal selective loss of peroxisomal enzymes and altered redox protein networks in MASLD, consistent with impaired pexophagy rather than generalized peroxisome depletion. Future strategies may combine pexophagy modulation with metabolic therapies. Proteomics-guided evaluation of peroxisomal proteome integrity may support rational combination approaches involving PPAR signaling and redox regulation [95].

## 7. Conclusions

Hepatocyte senescence has emerged as a central pathogenic driver of metabolic dysfunction–associated steatotic liver disease (MASLD), rather than a passive consequence of aging. Diverse stressors—including telomere dysfunction, DNA damage, oncogene activation, and mitochondrial impairment—converge on persistent DDR signaling and stable cell-cycle arrest. A key mechanistic insight from recent studies is that impairment of selective autophagy represents a critical interface between metabolic stress and hepatocyte senescence. Defective lipophagy, mitophagy, ferritinophagy, ER-phagy, and pexophagy lead to the accumulation of dysfunctional organelles, oxidative stress, and sustained DDR activation. Emerging proteomics-based evidence indicates that these defects are driven by coordinated remodeling of protein abundance, proteoform composition, and organelle-specific proteomes rather than by uniform transcriptional changes alone. However, most current studies rely on bulk or static measurements and do not adequately resolve cell-type-specific or spatial heterogeneity of senescent hepatocytes in MASLD. Collectively, these findings position selective autophagy and senescence as interconnected, proteomics-defined therapeutic targets in MASLD. Importantly, a major challenge moving forward is the lack of standardized, proteomics-based biomarkers that can distinguish adaptive versus maladaptive autophagy states and predict senolytic responsiveness. Future studies integrating quantitative, spatial, and proteoform-resolved proteomics with functional validation will be essential to define actionable targets. In particular, mapping organelle-specific proteome remodeling and autophagic flux across disease stages may enable stratification of MASLD patients based on senescence-associated protein signatures.

Thus, advancing a proteomics-centered framework will not only refine mechanistic understanding of hepatocyte senescence but also provide a foundation for precision therapeutic strategies targeting selective autophagy and proteostasis in liver disease. Importantly, the transition from MASLD to progressive inflammatory and fibrotic MASH appears to be closely linked to aging-associated decline in selective autophagy and accumulation of senescent hepatocytes. Therefore, therapeutic strategies aimed at restoring autophagic proteostasis and limiting senescence-associated proteome remodeling may represent promising approaches for preventing MASH progression and age-related liver dysfunction.

## Data Availability

No new data were created or analyzed in this study. Data sharing is not applicable to this article.
